# Adenomatoid Odontogenic Tumor Associated with an Impacted Maxillary Lateral Incisor: A Case Report with Five-Year Follow-Up

**DOI:** 10.1155/2017/1709492

**Published:** 2017-10-29

**Authors:** Najwa Karam Genno, Nicole Aoun, Sami El Toum

**Affiliations:** ^1^Department of Radiology, School of Dentistry, Lebanese University, Beirut, Lebanon; ^2^Department of Oral Pathology and Diagnosis, School of Dentistry, Lebanese University, Beirut, Lebanon

## Abstract

Adenomatoid odontogenic tumor (AOT), a benign (hamartomatous) lesion of odontogenic origin, is an uncommon tumor which affects mainly females in the second decade. This lesion is most commonly associated with an impacted maxillary canine. This paper reported a case of AOT, in a 16-year-old female, associated with an impacted maxillary left lateral incisor. The evolution of this tumor was followed over 36 months and 24 months after excision.

## 1. Introduction

Dreibaldt in 1907 was the first to describe adenomatoid odontogenic tumor (AOT), which is an uncommon benign epithelial lesion of odontogenic origin known as a pseudo-adenoameloblastoma [1]. The term “adenomatoid odontogenic tumor” proposed by Philipsen et al. [2] indicates that it was not a variant of ameloblastoma [2, 3]. In the World Health Organization classification of odontogenic tumors established in 1971, AOT was mentioned [1, 4] as a mixed odontogenic neoplasm, in other words, an epithelial tumor with an inductive effect on the odontogenic mesenchyme [1, 5].

It represents 3–7% of all odontogenic tumors, and over 750 examples have been reported in the literature. Some authorities feel that, given the slow growth and circumscription of the lesion, it is best classified as a hamartoma rather than a true neoplasm [3].

The adenomatoid odontogenic tumor is mostly limited to younger patients between 10 and 30 years [3, 6, 7], and two-thirds of all cases are diagnosed when the patient is 10–19 years old. This tumor is uncommon in a patient older than 30 years. It has a striking tendency to occur in the anterior portion of the jaws (95%) and is found twice as often in the maxilla (65%) than in the mandible. Females are affected about twice as often as males [3, 6, 8].

Most AOTs are relatively small. They seldom exceed 3.0 cm in greatest diameter, although a few large lesions have been described with a greater diameter of 7 cm [6]. Peripheral (extraosseous) forms of the tumor are also encountered but are rare [3, 8, 9].

AOT is frequently asymptomatic and is revealed during a routine radiographic examination or when radiographs are made to determine why a tooth has not erupted [3, 10]. A delayed eruption of a permanent tooth or a swelling of the jaws may be the first symptom [7]. Larger lesions cause a painless expansion of the bone [3, 8].

There are three variants of AOT. Follicular type (73%) has an intrabony lesion (central) associated mostly with an impacted tooth and is usually misdiagnosed as a dentigerous cyst or follicular cyst. Extrafollicular type (24%) has an intrabony lesion and no connection with the tooth. It is usually presented as a well-defined unilocular radiolucent image above or superimposed on the roots of the erupted teeth and often resembling a residual globulomaxillary or lateral periodontal cyst. Peripheral type (3%) usually presents as a gingival swelling and often appears as small, sessile masses on the buccal maxillary gingiva. Clinically, these lesions cannot be differentiated from the common gingival fibrous lesion [3, 4].

In about 75% of cases, the tumor appears as a circumscribed, well-defined unilocular radiolucency that involves the crown of an unerupted tooth, most often a canine which represents approximately 60% of cases. Permanent incisors, premolars, molars, and deciduous teeth are rarely involved [8, 10]. This follicular type of AOT may be impossible to differentiate radiographically from the more common dentigerous cyst. The radiolucency associated with the follicular type of AOT sometimes extends apically along the root past the cementoenamel junction. This feature may help to distinguish an AOT from a dentigerous cyst [3, 9, 10].

Microscopically, the tumor is composed of spindle-shaped epithelial cells that form sheets, strands, or whorled masses of cells in a scant fibrous stroma. The epithelial cells may form rosette-like structures around a central space, which may be empty or contain a small amount of eosinophilic material. This material may stain for amyloid [3, 10].

AOTs are benign and present a very low recurrence, making it unnecessary to carry out extensive and aggressive surgery [2, 3]. The surgical management of this lesion would be enucleation along with the associated impacted tooth because of its capsule; it enucleates easily from the bone [3].

In this paper, a case of AOT in the anterior maxillary region associated with a permanent lateral incisor will be reported. The evolution of this tumor was followed for 36 months before enucleation and 24 months after.

## 2. Case Report

**Figure 1:**
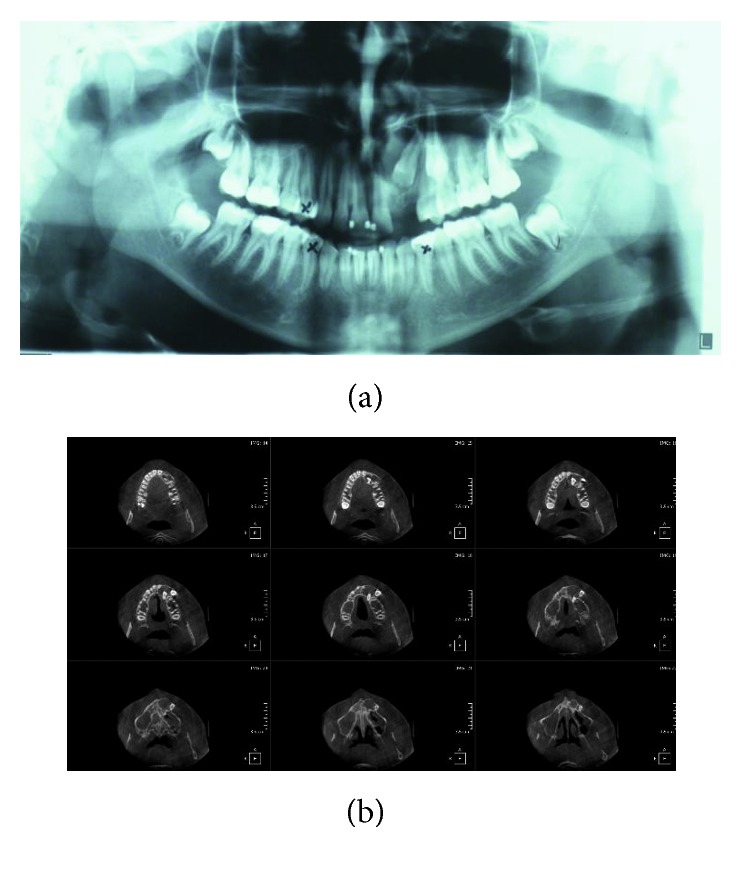
(a) Orthopantomogram and (b) axial view of cone beam computerized tomography showing a well-demarcated radiolucent image associated with impacted teeth #22 and 23.

A 16-year-old female consulted with a *chief complaint* of an asymptomatic swelling mass persistent from approximately 12 months on the left maxillary anterior region which increased gradually and achieved the present size of 1.5 cm. The history revealed an orthodontic treatment started in December 2011 with a treatment plan involving extraction of teeth #34, 44, 23, and 14. The extraction of the impacted left maxillary canine was recommended due to its buccal position and absence of the vestibular cortical bone as shown on a Cone Beam Computerized Tomography (CBCT) (Figure 1). A surgical exposition of the crown and bonding of a bracket on the maxillary left lateral incisor were also planned a coronoplasty of the first premolar to a canine shape. A radiolucency cyst-like was noted on the lateral incisor (Figure 1) and was diagnosed as a dentigerous cyst.

The extraction of the tooth #23 was done in April 2012, with surgery to bond a bracket on tooth #22, but it failed due to lack of the localization and the consistency of the tissues around the impacted tooth.

Her *medical observation* was noncontributory.

**Figure 2:**
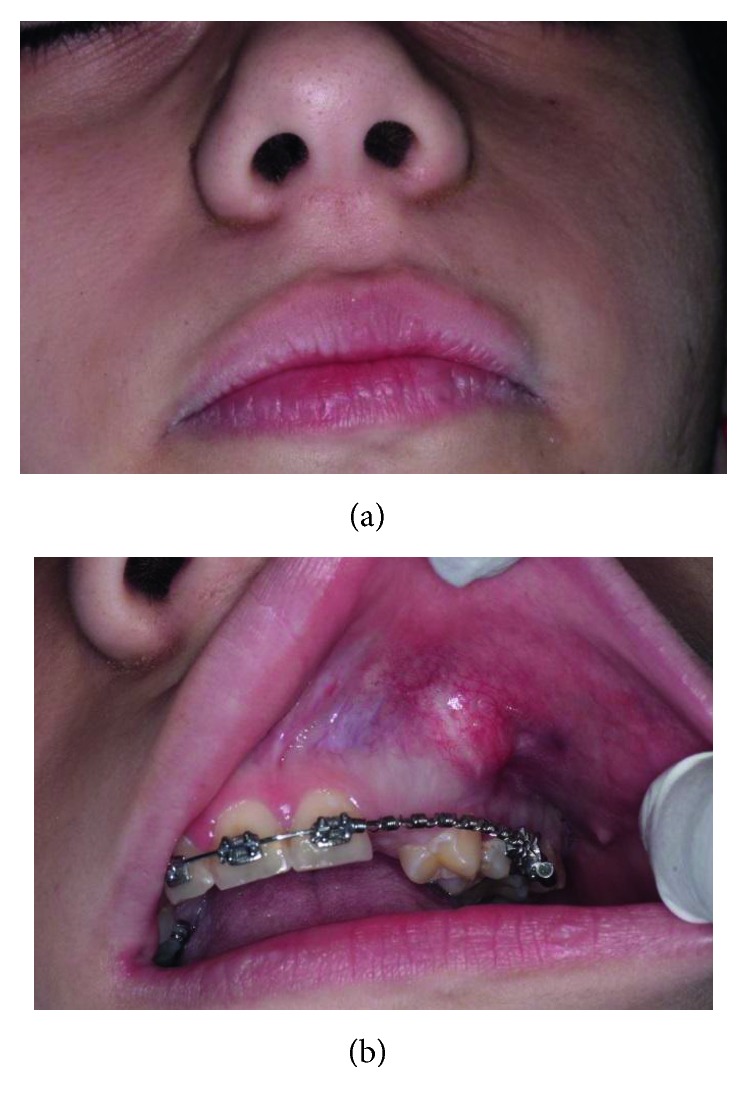
Clinical examination. (a) Extraoral view with asymmetry. (b) Intraoral view illustrating a swelling at the buccal fold.

On *extraoral examination* (Figure 2), facial asymmetry was noted. A solitary well-defined swelling on the left side of the face in the region of the nasal ala was palpable. The swelling was roughly oval, measuring approximately 2.5 cm in diameter, extending superoinferiorly from 0.5 cm above the ala of the nose to the midpart of the upper lip, mediolaterally from 0.5 cm lateral to the corner of the mouth to the nasolabial fold, causing mild asymmetry of the face. The skin over the swelling appeared normal. On palpation, the swelling was nontender, hard in consistency, and fixed to the underlying bone.

*Intraoral examination* (Figure 2) revealed a solitary smooth circumscribed swelling of 1.5 cm × 2 cm in size, with well-demarcated margins in the left maxillary region filling buccal vestibule. Anteroposteriorly, it extended from the mesial margin of the first premolar up to the distal margin of the second premolar. The buccal cortex was expanded, and the surface of the swelling was smooth with a normal color of overlying mucosa. The consistency was bony hard and nonfluctuant. On palpation, mild tenderness was present at one point.

The swelling was slow growing; it gradually increased in size and led to the disfigurement of the face.

Teeth examination revealed that teeth #22 and 23 are missing, and teeth #21 and 24 are positive at cold sensitivity test without mobility.

**Figure 3:**
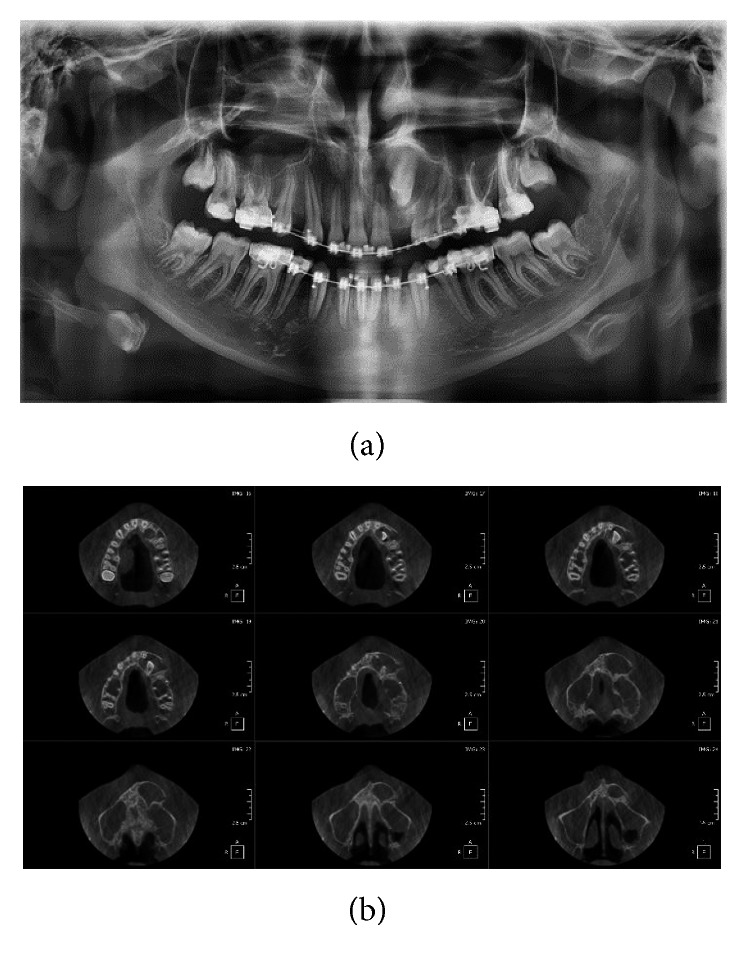
(a) Orthopantomogram and (b) axial view of cone beam computerized tomography, showing a well pericoronal circumscribed radiolucent image associated with impacted tooth #22.

*The axial slides of CBCT* (Figure 3) showed a well-circumscribed radiolucent lesion with well-defined radiopaque border extending horizontally from maxillary midline to the distal margin of the second premolar and vertically from the nasal base to the midpart of the roots of teeth #21 and 24. It is associated with an impacted lateral incisor. This radiolucency covers all crowns, and it overlaps the root of tooth #22.

Differential diagnosis of this image revealed a first dentigerous cyst, an adenomatoid odontogenic tumor, and a calcifying odontogenic cyst.

**Figure 4:**
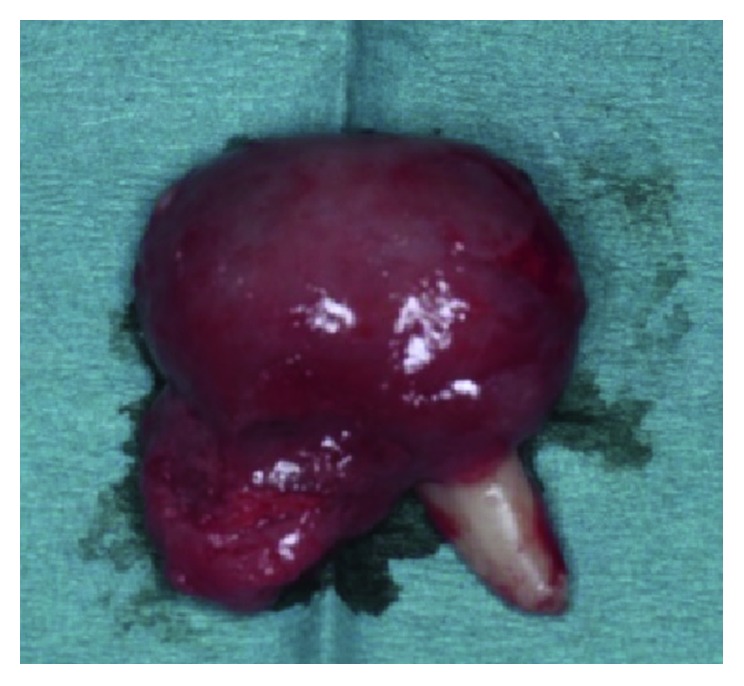
Enucleated mass with the root of tooth #22.

An enucleation was done. The tumor was well encapsulated, and the lateral incisor was easily removed with the lesion (Figure 4). The histopathologic examination confirmed the diagnosis of a follicular AOT type.

**Figure 5:**
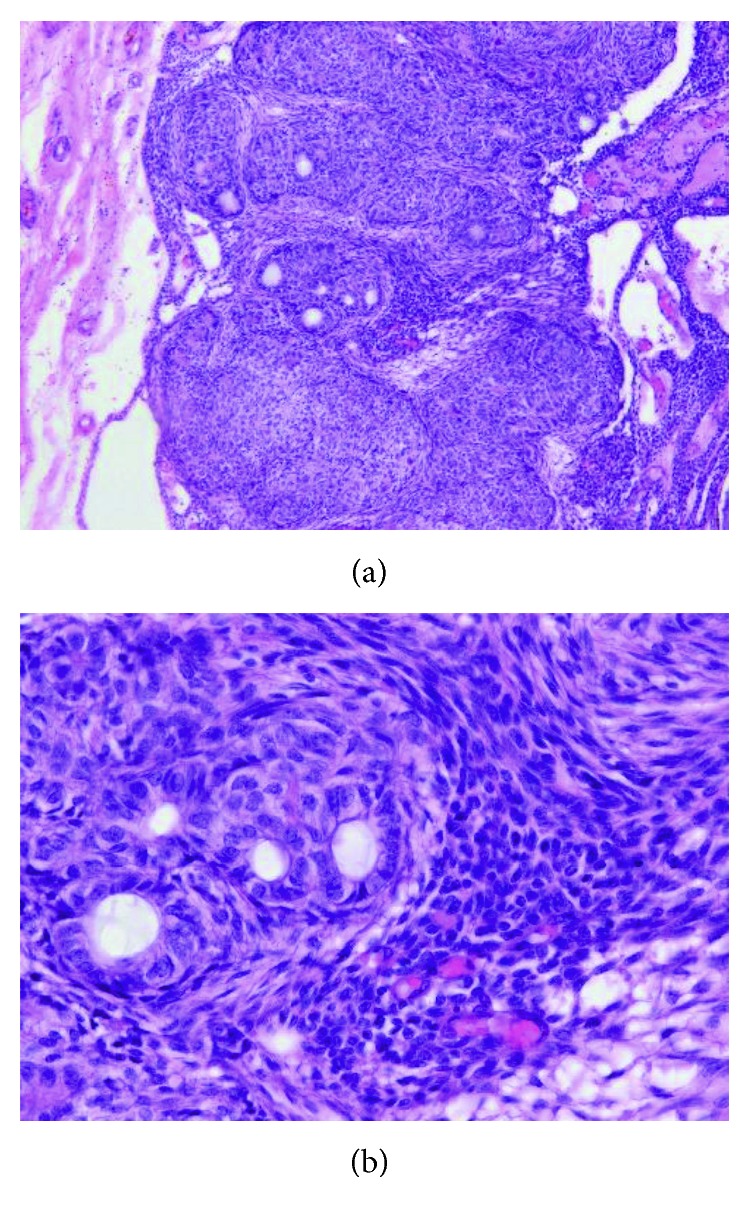
Adenomatoid odontogenic tumor. (a) Low-power view demonstrating a capsule surrounding the tumor. (b) Higher magnification showing the duct-like epithelial structures.

Microscopically, the tumor is composed of spindle-shaped epithelial cells that form sheets, strands, or whorled masses of cells in a scant fibrous stroma. The epithelial cells may form rosette-like structures around a central space, which may be empty or contain a small amount of eosinophilic material (Figure 5). This material may stain for amyloid.

**Figure 6:**
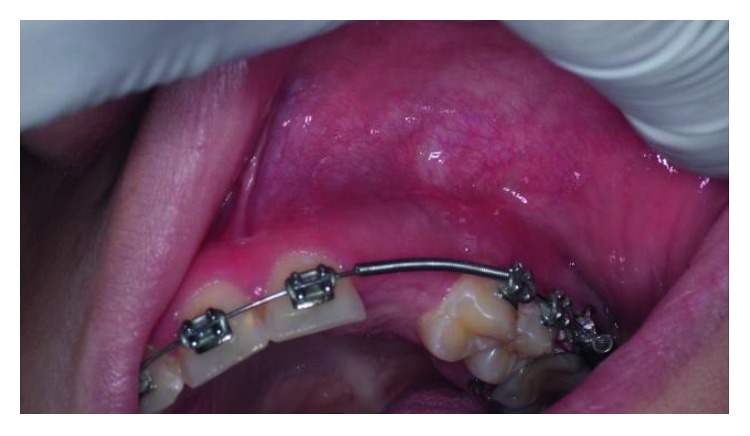
Two weeks postop.

**Figure 7:**
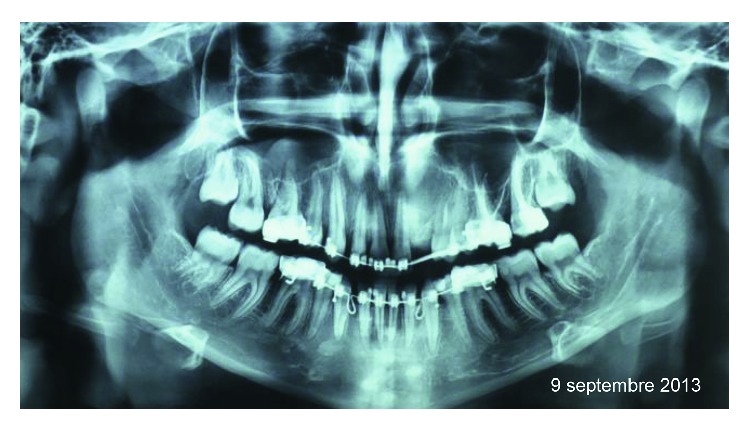
Orthopantomogram 3 months after surgery.

**Figure 8:**
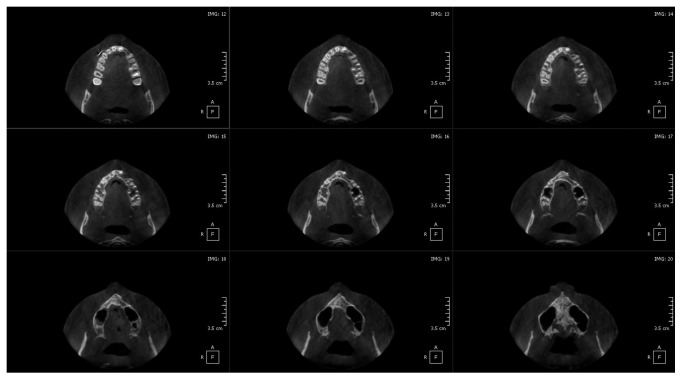
Axial view of cone beam computerized tomography showing the healing at the surgical site with the reduction of the bone width two years after surgery.

The healing was controlled for three weeks (Figure 6). The control orthopantomogram on the third month (Figure 7) followed by other on six months later showed normal bone trabeculation at the lesion site without recurrence. An orthopantomogram and CBCT were realized two years after for control, and placing an implant at the level of tooth #22 showed a reduction in the bone width in this region with complete bone healing (Figure 8).

## 3. Discussion

Mostly, nonneoplastic causes of jaw swelling in a young patient are an apical cyst, dentigerous cyst, calcifying odontogenic cyst, odontogenic keratocyst, and central giant cell granuloma [3, 10–12], whereas common neoplastic causes are an adenomatoid odontogenic tumor, unicystic ameloblastoma, calcifying epithelial odontogenic tumor (CEOT), ameloblastic fibroma, and ameloblastic fibro-odontoma [3, 12, 13].

A well-demarcated radiolucent lesion associated with the crown of impacted teeth like in this case ruled out the apical cyst, calcifying odontogenic cyst, odontogenic keratocyst, and central giant cell granuloma [10, 11, 14]. An apical cyst is usually associated with an endodontic-treated or mortified pulp tooth, but in this case, the teeth #21 and 24 were positive to cold sensitivity test. Calcifying odontogenic cyst, odontogenic keratocyst, and giant cell granuloma were ruled out because usually they are mostly not related to the crown of an impacted tooth, and they are mostly multilocular.

Dentigerous cyst, unicystic ameloblastoma, ameloblastoma, and ameloblastic fibroma are most frequent in the posterior region of the mandible and are associated in most cases with the third molar. However, adenomatoid odontogenic tumor occurs mostly in the anterior maxillary region and is associated in 60% of cases with a canine [3, 10, 14, 15].

A possible differential diagnosis for the lesion described in this case report is a dentigerous cyst and an adenomatoid odontogenic tumor.

Adenomatoid odontogenic tumor (AOT) is an odontogenic epithelial tumor [5, 14, 15] that is more commonly found in young female patients between 10 and 19 years of age. The maxilla is more commonly affected than the mandible. The size of the lesion ranges from 2 to 7 cm with a slow growing pattern which results in a painless expansion of the jaws. The canine is the most commonly impacted tooth [6, 10, 16]. In this case, a 16-year-old female presented with a well-demarcated radiolucent lesion of 2.5 cm diameter in the anterior maxillary region associated with an impacted permanent lateral incisor. The tumor was slowly growing within sixteen months; it filled the canine socket after extraction and expanded buccally to fill the buccal fold.

Mostly, AOTs are the central follicular type and appear as well-demarcated radiolucent lesions. AOT usually surrounds an unerupted tooth, and it looks as a corticated radiolucency with small radiopacities, but there are cases where the lesion has no radiopaque component, and in such cases, a dentigerous cyst is the preferred differential diagnosis. However, an AOT often appears to envelop the crown as well, where we divided the specimen to show the relation of the lesion to the tooth, unlike the dentigerous cyst which does not surround the roots [1, 3, 14, 16]. In this case, the lesion surrounded the entire crown and overlapped the root of the lateral incisor. Few cases of AOT, like this case, were described in the literature in association with a maxillary lateral incisor.

The histological typing of the WHO defined the AOT “like a tumor of odontogenic epithelium with duct-like structures and with varying degrees of inductive change in the connective tissue. The tumor is well encapsulated and shows an identical benign behavior” [1, 9, 16].

The treatment of choice was a surgical management of this tumor. It should be enucleated along with the associated impacted tooth and simple curettage [2, 6]. Conservative treatment is adequate because the tumor is not locally invasive, is well encapsulated, and can be easily separated from the bone. The surgical specimen may be solid or cystic. The recurrence rate is as low as 0.2%. However, in exceptional cases of large tumors or risk of bone fracture, partial resection, in a block of the mandible or maxilla, has been indicated. Also, the use of lyophilized bone and guided tissue regeneration are recommended in extended osseous cavities. The prognosis is excellent in the majority of the cases [2, 3, 10]. In this case, as described, enucleation was done with the impacted lateral incisor, which was removed with the lesion. The tumor is well encapsulated and covered the entire crown and overlapped the root of the lateral incisor. Follow-up 12–24 months after surgery was performed, and no recurrence was noted.

## 4. Conclusion

AOTs are usually asymptomatic lesions that sometimes may cause cortical expansion and displacement of the adjacent teeth; the slow growing nature of the lesion may cause the patient to tolerate the swelling for years until it produces an obvious facial deformity. Early diagnosis of this by the dental surgeon is mandatory when a clinical sign is mentioned, and early enucleation prevents an excessive destruction of bone. In this case, AOT was associated with the lateral maxillary incisor. The growth was slow over 18 months, which favors an early enucleation.
